# Thank you to *The Lancet Regional Health – Western Pacific’s* clinical and statistical peer reviewers in 2024

**DOI:** 10.1016/j.lanwpc.2025.101510

**Published:** 2025-02-28

Dmitry Abramov

Karen Adams

Demilade Adedinsewo

Alma J Adler

Sara Aghaee

Bernardo Aguilera

Bilal Ahmed

Linnea Aitokari

Seif Al Abri

Ziyad Al-Aly

Abdallah Al-Ani

Ali AlHamzawi

Gianfranco Alicandro

Sadeer Al-Kindi

Leopold Aminde

Anand Anbarasu

Craig Anderson

Ian P S Anderson

Gopinathan Anil

Suvaporn Anugulruengkitt

Jason Appleton

Nimalan Arinaminpathy

Dmitry Arkhangelsky

Elizabeth Armstrong

Saravanan Arunachalam

Hossam Ashour

Sumegha Asthana

Kostas Athanasakis

Katie Attwell

Abolfazl Avan

Sergey Avdeev

Suzanne R Avis

Shally Awasthi

George Ayala

Ganesh M Babulal

Helen Bailey

Paul Arthur Bain

Ram Bajpai

Roland Bal

Anindita Banerjee

Anyu Bao

Yanping Bao

Alfred Bartolucci

Krzysztof Bartuś

Gareth Baynam

Thomas Beaney

Amy Bei

Leila Bell

Yitayeh Belsti

Francisco J Benita

Sophie Bennett

Alberto Berardi

Michael Bergman

Johannes Berkhof

Ana Best

Zhao-xiang Bian

Melanie Birks

Nicolò Bizzarri

Joseph M Boden

Laura Botta

Sarah Bourke

Anders Boyd

Soren Brage

Eric Brunner

Danilo Buonsenso

Katrin Burkart

Eleanor Burnett

James Bussel

Zahid Butt

Xiaoling Cai

Yiyuan Cai

Marco Calabria

Claudia Calvano

Jackie Campbell

Sydney Campbell

Jun-Li Cao

Wei Cao

Yifan Cao

Yunlong Cao

Rachel Cassidy

Luca Ceccarelli

Xiangping Chai

Nathorn Chaiyakunapruk

Jason Yongsheng Chan

Polin Chan

Hsiao-Ting Chang

Wing Chung Chang

Xiao Chang

Tze-Fan Chao

Tony Charman

Hadrien Charvat

Emerson Chen

Hong-Lin Chen

Ingrid Chen

Renjie Chen

Runsen Chen

Ruoling Chen

Ruoqing Chen

Simiao Chen

Wanqing Chen

Wei Chen

Xiaoyuan Chen

Xinxin Chen

Yaolong Chen

Yihsing Chen

Yingyao Chen

Ying-Yeh Chen

Jian Cheng

Wei Cheng

Weibin Cheng

Xiaoshu Cheng

Ching-Lung Cheung

Ka Wang Cheung

Chi Leung Chiang

Pooja Chitneni

Kam-Lung Kelvin Chong

Mian Yoon Chong

Clara Chow

Dinh-Toi Chu

Brian Chung

Chen Chung-Yu

Vivienne Helaine Chuter

Camille Clare

Terryann Clark

Robert Clarke

Susan Clayton

Tammy Clifford

Marisa Cobanaj

Robert Colebunders

Caroline Colijn

Ian Colman

Alex Cook

Sarah Cook

Janine A Cooper

Andrew Copas

Roman Crazzolara

Sonya Cressman

Ernesto Crisafulli

Cory Cronin

Kristy Crooks

Matthew A Croxen

Ibrahim Dadari

Amarjargal Dagvadorj

Min Dai

Barbara Daly

Kelsey Dancause

Margie Danchin

Douglas R Danforth

Benjamin Daniels

Fabrizio D'Ascenzo

Soumitra Shankar Datta

Sarantsetseg Davaasambuu

Manuel Dayrit

Ruklanthi de Alwis

Geertruida de Bock

Jeroen De Bont

Lucie de Jonge

Dziedzom de Souza

Edward Dee

Bruno Degano

Anthony Delitto

Jonas Demant

Salil Deo

Alexandra Descarpentrie

Andrew Dessler

Michael Dewey

Shyamali Dharmage

Qian Di

Massimo Di Maio

Julio Diaz

Joseph Dieleman

Peter Diggle

Joakim Dillner

Song-Ze Ding

Tao Ding

Kim N Dirks

Lien Anh Ha Do

Matthew Dodd

Richard Dodel

Jianzeng Dong

Shuhan Dong

Yaa-Hui Dong

Gregory Dore

Dung Do-Trung

Nicholas Douglas

Abigail Dove

Lingbin Du

Xiaoxing Du

Charmaine Duante

Ann-Sofi Duberg

Jaime Duffield

Andre Duraes

Ralf Dürrwald

Lara Edbrooke

Asmaa El-heneidy

Jonathan Emberson

Connie Erkens

Mohammed Eslam

Paolo Eusebi

D Gareth Evans

Daniel John Exeter

Gian Paolo Fadini

Lei Fan

Chi-Tai Fang

Hai Fang

Eric Faragher

Forough Farrokhyar

Louis Favril

Luzhao Feng

Zhongxiang Feng

Javier Fernández De Cañete

Alize J Ferrari

Natan Feter

Suzanne Filteau

Florian Fischer

Katja Fischer

Robert Fitridge

Joël Fokom Domgue

Chuan De Foo

ChingChing Foocharoen

Florence Fouque

Nicola D Foxlee

Anna Freni Sterrantino

Gengfeng Fu

Hongqiao Fu

Lijun Fu

Ping Fu

Shau-Huai Fu

Jens Fuglsang

Taiyo Fukai

Kamil Fuławka

Albis Francesco Gabrielli

Kurubaran Ganasegeran

Wei Gao

Zhongshan Gao

Tushar Garg

Long Ge

Yihui Ge

Cindy George

Evangelos Giamarellos-Bourboulis

Alberto Giannoni

Duha N Gide

Amit Goel

Anne Eng Neo Goh

Debora Goldberg

David Goldsbury

Delia Goletti

Jessica Gong

Wenjie Gong

Jan Gothard

Jiangtao Gou

Andrew Gray

Martin Grobusch

Dongmei Gu

Zhi–Chun Gu

Thomas Guadamuz

Xiaodong Guan

Vivian Yawei Guo

Luis Miguel F Gutiérrez-Robledo

Assad Hafeez

Bridget Haire

Michaela Hall

Masahide Hamaguchi

Chisato Hamashima

Najmeh Hamzavi-Zarghani

Cheng Han

Tianyu Han

Chuangli Hao

Hideyuki Harada

Billie Jo Hardy

Bernhard Haring

Patrick Harris

Danielle L Harrop

Mia Hashibe

Hideki Hashimoto

Marie Hauguel-Moreau

Markus Haun

Cheng He

Dalin He

Ping He

Tianfeng He

Yulei He

Jeong Heo

Seulkee Heo

Neil J Hime

Sameer A Hirji

Iris Szu-Szu Ho

Thang Hoang

Valerie Hoffman

Thomas Hofmarcher

Hans Hogerzeil

Anna-Clara Hollander

Preben Homøe

Joanna Hope

Kara K Hoppe

Fabienne Hornfeck

Ahmed Hossain

Ningning Hou

Xiangyu Hou

Gerard Hoyne

Cyrus Hsia

Chien Ning Hsu

Dongsheng Hu

Fupin Hu

Hao Hu

Peng Hu

Huimin Huang

Jee-Fu Huang

Poyin Huang

Yueqin Huang

Jimi Huh

Yong Il Hwang

Carolina Ibáñez

Moritake Iguchi

Fusao Ikawa

Rintaro Imafuku

Arkaitz Imaz

Jose M Inoriza

Ken Inoue

Yosuke Inoue

Masao Iwagami

Christopher Jackson

Tazeen Jafar

Ram Jagannathan

Edward Janus

Heidi Janz

Luis Jara-Palomares

Kristina Jenei

Adam W J Jenney

Signe Jervelund

Sophie J Jessop

John Ji

Cun-Xian Jia

Jianping Jia

Na Jia

Zhongwei Jia

Quanbao Jiang

Xinyi Jiang

Yawen Jiang

Ane Johannessen

Bernadette Jones

Kristina Jönsson

Gabby B Joseph

Paul Joyce

Steven Julious

Jiyun Jung

Zubair Kabir

Shigenori Kadowaki

Joseph Keawe‘aimoku Kaholokula

Zihan Kan

Jihun Kang

Md Nazmul Karim

Takatoshi Kasai

Naoki Kashihara

Lewis E Kazis

Ramesh Kekunnaya

Charlotte Kelley Jones

Mark Kelson

Zoe Kelson

Alex R Kemper

Syed Afroz Keramat

Ali Khashan

Resham Khatri

Panarasri Khonputsa

Zi Xean Khoo

Yet Khor

Haitham Khraishah

Don Osami Kikkawa

Bo Kim

Bongyoung Kim

Seok Jin Kim

Sung-Bae Kim

Tae Hyun Kim

Won Kim

Wookun Kim

Jonathan King

Tania King

Christa Koenig

Cristian Koepfli

Sebastian Kohlmann

Kentaro Koide

Kairi Kolves

Evangelos Kontopantelis

Marko Korhonen

Caroline Kramer

Evangelos Kritsotakis

Kishore R Kumar

Ho-Chang Kuo

Hannah Kuper

Ada Kwan

Yat wah Kwan

Yu heng Kwan

Lies Lahousse

Chih-Cheng Lai

Yun-Ju Lai

Arush Lal

Sham Lal

Matteo Lambertini

Manyu Lan

Tyler Lane

Peter Lange

Wallis Lau

Erika J Laukka

Carl Lavie

Payton Lea

Choung Ah Lee

Hocheol Lee

Ji-ho Lee

Jung Eun Lee

Keunhwa Lee

Tetz Cheng-Che Lee

Victor Ho-Fun Lee

Wen-Chung Lee

Whanhee Lee

Whanhee Lee

Yo Han Lee

Yu-lin Lee

Borwornsom Leerapan

Maud Lemoine

Lea Lenglart

Stuart Leske

Jodie Leu

Angela YM Leung

Nicolas Leveziel

Zohar Levi‬‏

Ang Li

Ben Li

Chao Li

Guangdi Li

Guanqiao Li

Huai-chen Li

Jianghong Li

Li Li

Lin Li

Liping Li

Mengfeng Li

Ni Li

Ning Li

Philip K T Li

Qihan Li

Ruichao Li

Sheyu Li

Taisheng Li

Tiantian Li

Wei Li

Wendy Li

Xi Li

Xin Li

Yan Li

Yang Li

You Li

Zixiao Li

Jian-Qi Lian

Lee-Ling Lim

Seng Gee Lim

Giuseppe Limongelli

Chien Yu Lin

Chunqing Lin

Jialing Lin

Ke Lin

Kuanyin Lin

Joni Lindbohm

Paul S Links

Lynda Lisabeth

Bao-Peng Liu

Chaojie Liu

Chenxi Liu

Chun-Jen Liu

Cong Liu

Jinhui Liu

Jinli Liu

Jue Liu

Jufen Liu

Junqi Liu

Longding Liu

Min Liu

Po-Yu Liu

Tao Liu

Xuxiang Liu

Yong Liu

Yuewei Liu

Zheng Liu

Roanna Lobo

Michael James Loftus

Samantha Loi

Zerina Lokmic-Tomkins

Jorge Lopez-Castroman

Kevin Lu

Xiaoxia Lu

Yingli Lu

Zhihao Lu

Juliana Lui

Andrea Luk

Tom Lung

Hao Luo

Xingxian Luo

Jun Lv

Amy D Lykins

Frances Lynch

Changsheng Ma

Edmond Shiu Kwan Ma

Jun Ma

Tianyi Ma

Stephen Macfarlane

Catherine MacPhail

Lina Madaniyazi

Rodolphe Mader

Hideki Maeda

Fabrizio Maggi

Martina Anna Maggioni

Royford Magiri

Mathieu Maheu-Giroux

Mohammad Afzal Mahmood

Maria Ida Maiorino

Sandawana William Majoni

Stephen David James Makin

Christoph Male

Rahul Malhotra

Andrei Malinovschi

Shumei Man

David M Mannino

Mohammad Ali Mansournia

Jennifer Manuel

Yimin Mao

Ulrich Marcus

Eloi Marijon

Marianne Martinello

Javiera Martinez-Gutierrez

Sri Masyeni

Kentaro Matsui

Martin E Matsumura

Fiona Matthews

Christophe Matthys

Juliette H Mazereeuw-Hautier

Mikael Mazighi

Placide Mbala

Tim McCreanor

Jacqueline McIntosh

Conor Meehan

Kirsten Mehlig

Erik Meijer

Maria Melchior

Bijoy Menon

Fiona Mensah

Leilani Mercado-Asis

Benjamin J Metcalf

Ricarda Mewes

Daniel Michaeli

Jo Middleton

Keisuke Miki

Steven M Miller

Iona Millwood

Satoru Miyawaki

Daisuke Mizushima

Ashraf Mohammadkhani

Masoud Mohammadnezhad

Devi Mohan

Viswanathan Mohan

Sanjay Mohanty

Javadpour Mohsen

Mashkoor Mohsin

Chris Ka Pun Mok

Miguel Angel Molina-Vila

Mark Molitch

David Moore

Robert Moran

Masaaki Mori

Chloe Morozoff

Mohammad Izzat Morshidi

Rami Morsi

Jeremias Motte

Nor Muhamad

Michio Murakami

David Murdoch

Narcisa Muresu

Shigeo Muro

Anne Margrethe Myhre

Woojae Myung

Misako Nagasaka

Nicolas Nagot

Hiromichi Naito

Ryo Naito

Miharu Nakanishi

Taizen Nakase

Steven Narod

Anne Neilan

Carmen Ng

Fiona Ng

Daniel Ngui

Minh Nguyen

Thu-Anh Nguyen

Hai Nguyen-Tran

Sheng Nie

William D Nikolakis

Qin Ning

Dipesh Niraula

Shuhei Nomura

John Norrie

Kathleen O'Brien

Eric Ohuma

Toshiyuki Ojima

Motohiro Okada

Kentaro Okamoto

Kazuya Okamura

Tomonori Okamura

Ikechi Okpechi

Andre Oliveira

Dominic Oliver

Rianne Oostenbrink

Johanna Ospel

Afshin Ostovar

Megan Othus

Chun-Quan Ou

Kevin Outterson

Sean Owens

Alicia Padron-Monedero

Joseph Palamar

Raffaele Palladino

Christopher Palmer

Fen Pan

Jay Pan

Jia Wern Pan

Xiong-Fei Pan

Junxiong Pang

Yuanjie Pang

Leonardo Pantoni

Dimitrios Paraskevis

Elena Pariani

Jean-Jacques Parienti

Donald Parsons

Thaigarajan Parumasivam

Imre Pávó

Lillian Pazvakawambwa

Madeline Pe

Astrid Pechmann

Marijke Peetermans

Sarah Pendlebury

Junping Peng

Songxu Peng

Jose Perez-Molina

Martin Pfeffer

Lily A Pham

Roy Philip

Arthit Phosri

Alessandra Pierangeli

Alessandro Pileri

Sara Pilotto

Farhad Pishgar

Carlo Andrea Pivato

Jonathan M Platt

May Ee Png

Kian Keong Poh

Paul Poirier

M Cristina Polidori-Nelles

Constance Dimity Pond

Emanuele Pontali

Kathleen M Potempa

Robin Prescott

David Prior

Christy Pu

Milo Puhan

Marianna Purgato

Shunan Qi

Shangshang Qin

Xianhui Qin

Kieran Quinn

Terence Quinn

Huma I Qureshi

Jeyamani Ramachandran

Reshma Ramachandran

Jacqueline Ramke

Thais Rangel Bousquet Carrilho

Caitlin Rausch

Devin Razavi-Shearer

Andrea Rehman

Rachel Reilly

Nice Ren

Ana Ribeiro

Alan S Rigby

Eli Ristevski

Kris Rogers

Damian T Roland

Anna K Rolleston

Rafael Costa Rosell

Pierre-Marie Roy

Dominic Royé

Yibing Ruan

Salvatore Rudilosso

Yun-Feng Rui

Jose Ignacio Ruiz Azpiazu

Mencía Ruiz Gutiérrez-Colosía

Soomin Ryu

Michela Sabbatucci

Marc Saez

Dodi Safari

Ranjit Sah

Sabine Salloch

Mario Salvi

José Sanches

Pruthi Sandhya

Pekka Olavi Santtila

Tailane Scapin

Axel Schmidt

Sneha Annie Sebastian

Andrea Sechi

Menno W Segeren

Carlo Senore

Helena Seth-Smith

Prakash Shakya

Guangliang Shan

Xianwen Shang

Ying Shang

Kamal H Sharma

Monisha Sharma

Namrata Sharma

Phil L Shearman

Mahdi Sheikh

Chi Shen

Yongchun Shen

Han-Ping Shi

Qiuling Shi

Weifeng Shi

Yoko Shibata

Yusuke Shimakawa

Kazuki Shimizu

Takayuki Shimizu

Yuki Shirakura

Marcelo Silva

Hope Simpson

Kritsada Singha

Daisy Singla

Miri Levi Sklair-Levy

Brian M Slomovitz

David Smith

Winnie So

Anna Solini

Erwei Song

Weihong Song

Milan Sonneveld

João Andre Sousa

Nina R Sperber

Christian Staerk

Jannik Stemler

Scott Stevens

Mark Stokes

Wilma Stolk

Christopher Su

Guobin Su

Zhaohui Su

Elena Succurro

Rhiann Sue See

Yi Nam Suen

Shuichi Suetani

Yi Sui

Baoqing Sun

Dianjianyi Sun

Guiju Sun

Hsin-Yun Sun

Kai Sing Sun

Qiang Sun

Maria Sundaram

Fung-Chang Sung

Sopak Supakul

Diya Surie

Anna Szücs

Ozra Tabatabaei-Malazy

Masaya Takahashi

Yoshihiro Takashima

Koichi Takayama

Nanako Tamiya

Ryota Tamura

Benjamin Tan

Edwin Tan

Jackson Tan

Kit-Aun Tan

Maw Pin Tan

Ngiap Chuan Tan

Wei Long Tan

Atsushi Tanaka

Tetsuhiro Tanaka

Jie Tang

Nai-jun Tang

Shenglan Tang

Wenxi Tang

Xun Tang

Yi-Da Tang

Dan Tao

Alvin Kuowei Tay

John Patrick Taylor

Chikashi Terao

Yasuo Terauchi

Mary Beth Terry

Yih-Chung Tham

Sathish Thirunavukkarasu

Chloe Thomas

Katherine Thurber

Erik Timmermans

Jill Tinmouth

Eirik Brekka Tjonnfjord

Michael Tong

Yongsheng Tong

Harrys Torres

Marcos Roberto Tovani-Palone

Janet Towns

Kazunori Toyoda

Nu Quy Linh Tran

Pascart Tristan

Tobias Tritschler

Ming Horng Tsai

Ka Yu Tse

Kazuya Tsubouchi

Akizumi Tsutsumi

David Tunnicliffe

Barbara Turner

Veronica Ueckermann

Sean Urwin

Ramyadarshni Vadivel

Bouchez Valerie

Stijn van der Veen

Willem A Van Gool

Folkert van Kemenade

Daniela van Santen

Ravipa Vannakit

Maarten Vermeer

Lasse Vestergaard

Frank Visseren

Luan Nguyen Quang Vo

Hedwig Vos

Angela Waanders

Jonas Wachinger

Stephen Wade

Imam Waked

Kayo Waki

Susan Walker

Hannah Wallace

Susan Waller

Chris Wallis

Melissa Walls

Chengdi Wang

Chongjian Wang

Huali Wang

Jianming Wang

Jiguang Wang

Ninglan Wang

Qian Wang

Qiang Wang

Qiang Wang

San Ming Wang

Shi-Heng Wang

Shuhang Wang

Xiaoli Wang

Xiaoping Wang

Xuemei Wang

Yaogang Wang

Yong-Fei Wang

Yuan Yuan Wang

Yuanyuan Wang

Zhe Wang

Zhizhong Wang

Zixin Wang

Kinley Wangdi

Mary Njeri Wanjau

Laura Waters

Marianne Frances Weber

Brendan S Weekes

Liu Wei

Yongyue Wei

Paul Weiss

Heiman Wertheim

Anthony White

Anna Wilkinson

Iestyn Williams

Barne Willie

Stefan Willmann

Pattanee Winichagoon

Rebecca Winter

Ferdinand Wit

Charles Shey Wiysonge

Eliza L Y Wong

Martin Wong

Jean Woo

Chao Wu

Chi Shin Wu

Chunmei Wu

Hongjiang Wu

Jianyong Wu

Jinhong Wu

Liuliu Wu

Rui Wu

Tongzhi Wu

Zekai Wu

Dan Xiao

Xiang Xiao

Yonghong Xiao

Geng Xu

Jian Xu

Jianmin Xu

Jiayuan Xu

Lingzhong Xu

Liyue Xu

Min Xu

Pingyi Xu

Richard Huan Xu

Wanghong Xu

Weize Xu

Yang Xu

Zhiwei Xu

Ling Xue

Uday Yadav

Gavin Yamey

Aimin Yang

Chao Yang

Grace Meijuan Yang

Huafeng Yang

Jun Yang

Lejin Yang

Qingwu Yang

Ting Yang

Xueli Yang

Yue Yang

Yao Yao

Kazuo Yashima

Pengpeng Ye

Steven Yeh

Su-Peng Yeh

Saeed Yekaninejad

Leonard Yeo

Yongmei Yin

Naohiro Yonemoto

Yoshikuni Yoshida

Hua You

Seng Chan You

Doris Young

Bin Yu

Bin Yu

Ruichuan Yu

Yunsong Yu

Zhebin Yu

Jing Yuan

Justin Zachariah

Wan Mohd Zahiruddin

Rabaab Zahra

Alberto Zambelli

Dorota Zarębska-Michaluk

Jean Pierre Zellweger

Jinhao Zeng

Xiaoxi Zeng

Yan Zeng

Shixian Zhai

Zhenguo Zhai

Fengqing Zhang

Huabing Zhang

Jianjun Zhang

Lei Zhang

Shihui Zhang

Shouzi Zhang

Shuyang Zhang

Xiaomin Zhang

Yue Zhang

Zhu Zhang

Baihui Zhao

Chongbo Zhao

Dong Zhao

Guohua Zhao

Jian Zhao

Shi Zhao

Yang Zhao

Ming-Hua Zheng

Xiaoying Zheng

Bolun Zhou

Hua Zhou

Huifang Zhou

Jiansong Zhou

Kaixin Zhou

Rongrong Zhou

Wangda Zhou

Zhemin Zhou

Zhen Zhou

Zijian Zhou

Bin Zhu

Wei Zhu

Zhuoting Zhu

Guihua Zhuang

Chao Zhuo

Zhiyong Zong

Zhiyong Zou

Gianvincenzo Zuccotti

Sa’ed H Zyoud

